# Copper/Carbon Core/Shell Nanoparticles: A Potential Material to Control the Fish Pathogen *Saprolegnia parasitica*

**DOI:** 10.3389/fvets.2021.689085

**Published:** 2021-07-23

**Authors:** Jv Zhang, Juncai Chen, Qianjun Huang, Brett MacKinnon, Omid Nekouei, Hong Liu, Peng Jia, Jinjin Wang, Na Li, Liqing Huang, Ying Yang, Pok Ng, Sophie St-Hilaire

**Affiliations:** ^1^Department of Infectious Diseases and Public Health, Jockey Club College of Veterinary Medicine and Life Sciences, City University of Hong Kong, Hong Kong, China; ^2^State Key Laboratory of Aquatic Animal Health at the Animal and Plant Inspection and Quarantine Technical Centre, General Administration of Customs, Shenzhen, China; ^3^Animal and Plant Inspection and Quarantine Technical Center, Shenzhen Customs District, Shenzhen, China; ^4^Shenzhen Technology University, Shenzhen, China

**Keywords:** *Saprolegnia parasitica*, metal-based nanoparticles, CCCSNs, filter, water disinfection

## Abstract

Copper-based fungicides have a long history of usage in agriculture and aquaculture. With the rapid development of metal-based nanoparticles, copper-based nanoparticles have attracted attention as a potential material for prevention and control of *Saprolegnia parasitica*. The present study investigated the effectiveness of copper/carbon core/shell nanoparticles (CCCSNs) and a commercial CCCSNs filter product (COPPERWARE^®^) against *S. parasitica* in a recirculating system. Results showed that the growth of agar plugs with mycelium was significantly suppressed after exposure to both CCCSNs powder and COPPERWARE^®^ filters. Even the lowest concentration of CCCSNs used in our study (i.e., 100 mg/mL) exhibited significant inhibitory effects on *S. parasitica*. The smallest quantity of the filter product COPPERWARE^®^ (3.75 × 3.7 × 1.2 cm, 2.58 g) used in our aquarium study also demonstrated significant inhibition compared with the control group. However, we observed leaching of copper into the water especially when larger quantities of COPPERWARE^®^ were used. Water turbidity issues were also observed in tanks with the filter material. Besides these issues, which should be further investigated if the product is to be used on aquatic species sensitive to copper, CCCSNs has promising potential for water disinfection.

## Introduction

Saprolegniasis is one of the most prevalent oomycete diseases in aquaculture (1), and among the Saprolegnia species, *Saprolegnia parasitica* is an important pathogen of finfish (2). The historical treatment for saprolegniasis was malachite green; however, this substance is now banned for use in food fish in many countries due to its teratogenic and mutagenic properties (3). Formalin is now the most commonly used treatment for this pathogen (4), but it also has potential carcinogenic and allergenic properties (5). Formalin treatments are forbidden in some countries (6) and more jurisdictions are expected to follow (7). Several other treatments such as salt (8), bronopol (9), and ozone (10) have been reported to be somewhat effective against *Saprolegnia* spp. However, their use is limited due to either adverse impacts on the environment or the aquatic animals, or limited efficacy. For example, although prolonged salt immersion is effective to inhibit *S. parasitica*, this treatment is impractical in large freshwater systems (11). Bronopol is effective against saprolegniosis (7), but tolerance to this product has been reported (12). Powerful oxidants such as ozone and hydrogen peroxide can reduce *Saprolegnia* spp., but these may also damage the gills of fish (13, 14). More recently potential inhibitors of *S. parasitica* such as triclosan and azelaic acid have been identified, however, their practical application needs further investigation (15). Despite numerous attempts to find safer, more effective, and environmentally friendly alternatives to malachite green, the solution to controlling saprolegniasis is still elusive (1, 4).

Metal-based nanoparticles with particle sizes <100 nm are being explored as an alternative approach to control infectious diseases caused by pathogenic bacteria (16, 17) and fungi (18, 19). These novel materials have distinct physical and chemical properties (20), different from their bulk counterparts or molecular compounds, that enhance bacterial binding, disruption of cell membranes, inhibition of enzyme activity and DNA synthesis (21, 22). Their promising results have raised interest in evaluating metal-based nanoparticles in the field of aquaculture to reduce pathogens in the water (23–28).

Copper-based nanoparticles are of particular interest in aquaculture because copper-based chemicals have long been used to control algal growth (29, 30), parasites, and saprolegniasis (31–33). For example, traditional copper sulfate was reported to prevent winter kill from occurring in over 90% of the fish before the water mold infection was visible on the fish (34). Copper-based nanoparticles have an increase in surface area to volume ratio, which provides them with a large biological active surface and may improve the efficacy of copper (35). These compounds are starting to be recognized as having a wide range of antifungal and antibacterial properties that could be used in agriculture (36), and in the biomedical field (37–39).

Copper-based nanoparticles have been reported to display better inhibition of fungus relative to other nanoparticles (40). However, the use of copper-based nanoparticles in aquaculture, has focused on the control of aquatic bacteria (41, 42), with limited information on their use for controlling oomycete pathogens such as *S. parasitica*. Copper is cost-effective relative to other metals such as silver, and therefore may be more suitable for low-cost large-scale water disinfection; filters made from these materials may be adapted relatively inexpensively to existing filter systems to reduce fungus-like agents in aquaculture settings.

Copper/carbon core/shell nanoparticles (CCCSNs) are a type of copper-based nanoparticles coated with a thin protective carbon shell (27). This coating is suppose to reduce the amount of copper ions released into the environment. Maintaining copper in a bound state is important in aquaculture as some species of fish, and shrimp do not tolerate exposure to high levels of copper (43, 44). In addition, metallic nanoparticles without a protective coating often have a high propensity to oxidize or undergo other chemical reactions (45). It has been speculated that the core-shell structure of CCCSNs may prevent copper from dissolving into the environment and protect it against chemical reactions, which could provide an environmentally friendly and longer lasting approach to control *S. parasitica* and other microorganisms in water.

The aims of this study were to: (1) investigate the efficiency of CCCSNs against *S. parasitica*; (2) evaluate a commercial CCCSNs filter product (COPPERWARE^®^) in reducing *S. parasitica* in a recirculating aquarium system; and (3) evaluate the effect of the COPPERWARE^®^ on water quality parameters (copper concentration, turbidity, pH, and dissolved oxygen).

## Materials and Methods

### Purification of *Saprolegnia parasitica*

A *Saprolegnia parasitica* isolate stored at −80°C at the State Key Laboratory of Aquatic Animal Health at the Animal and Plant Inspection and Quarantine Technical Centre, in Shenzhen Customs District, General Administration of Customs, P. R. China was streaked on solid potato dextrose agar (PDA) medium (Land Bridge Co., Ltd., Beijing, China) for 3 days at 25°C. Subsequently, an agar plug was cut from the PDA medium and used in our experiments.

### CCCSNs Powder on the Growth of Mycelium

The anti-oomycete activity of CCCSNs (Suzhou Guanjie Technology Co., Ltd., China) was examined by evaluating the growth of *S. parasitica* on PDA with different concentrations of CCCSNs (1, 5, 10, 50, 100, 500, 1,000, 1,500, 2,000 mg/L). In brief, CCCSNs were added to PDA while it was in liquid form and sonicated (100 W, 40 kHz) for 30 min under 60°C to increase the dispersion of the nanoparticles (46). The mixture was poured into petri plates. There were three replicates for each concentration including the negative control, which had no CCCSNs. An 8 mm diameter agar plug with mycelium growth was placed in the center of each plate. Cultures were incubated at 25°C and the growth of the hyphae was measured after 24, 48, 72, and 144 h. The colony diameter of the growing mycelium was determined by averaging two measurements taken at 90° from each other. We compared the growth of the mycelium plugs at different concentrations of CCCSNs over time as explained in section Statistical Analyses.

### CCCSNs Filter (COPPERWARE^®^) Filtration Experiments

After we confirmed the minimum effective concentration of CCCSNs on the mycelium growth of *S. parasitica*, a commercial filter containing CCCSNs branded as COPPERWARE^®^ (Suzhou Guanjie Technology Co., Ltd., China) was included in aquarium filters to assess the water-disinfection properties of the filter product. We conducted two independent experiments (experiment 1 and experiment 2) to assess the anti-oomycete property of different quantities of COPPERWARE^®^ as described in Table 1. During these studies, we also measured changes in water quality (pH, dissolved oxygen, turbidity, ammonia, nitrite, nitrate, copper) over time. Lastly, at the end of experiment 2, we assessed whether *S. parasitica* had the ability to rejuvenate when transferred to an environment without CCCSNs.

**Table 1:** Description of the parameters used in the COPPERWARE^®^ filtration experiments (experiment 1 and experiment 2).

**Experiment**	**Duration**	**Fungus size**	**Different sizes of COPPERWARE^^®^^^a^**
Experiment 1	72 h	8 mm	L-copperware: 7.5 × 3.7 × 2.4 cm, 9.46 g M-copperware: 7.5 × 3.7 × 1.2 cm, 4.96 g No-copperware: filter without CCCSNs
Experiment 2	144 h	5 mm	M-copperware: 7.5 × 3.7 × 1.2 cm, 4.96 g S-copperware: 3.75 × 3.7 × 1.2 cm, 2.58 g No-copperware: filter without CCCSNs

a *L, large; M, medium; S, small; No, no CCCSNs in the filter*.

#### Experiment 1

In experiment 1, we compared two quantities of COPPERWARE^®^ (see Table 1 for quantities) on the growth of *S. parasitica* for a duration of 72 h. In brief, nine aquariums (three with the high quantity of COPPERWARE^®^, labeled with L-copperware; three with a medium quantity of COPPERWARE^®^, labeled with M-copperware; and three control tanks, labeled with No-copperware) with a volume of 5 L were filled with 4.98 mL of deionized water. An 8 mm PDA agar plug colonized with *S. parasitica* and 22 mL of potato dextrose broth were added to each aquarium. Continuous circulation through the filtration system (SZ-230A, Jeneca, China) was administered during the experiment. At the end of the experiment (72 h), agar plugs from each tank were removed and the colony diameters were measured by averaging two measurements taken at 90° from each other. We compared the growth of the agar plug between treatments over time (refer to section Statistical Analyses). Microscopy (AXio Imager M1, ZEISS, Germany) was used to inspect the morphology of *S. parasitica* at the end of the trial.

#### Experiment 2

In experiment 2, we compared two different sizes of COPPERWARE^®^ filter material. In this experiment three tanks were set up with a medium quantity of COPPERWARE^®^ labeled M-copperware and three tanks had a small quantity of COPPERWARE^®^ labeled S-copperware. We also had three tanks with the filter material, but no CCCSNs for comparison. An 5 mm agar plug size was added to the tanks and monitored for a duration of 144 h. The anti-oomycete efficiency of the material was assessed in a similar manner as described in experiment 1.

### Post-effect of COPPERWARE^®^ on *S. parasitica* Survival

**Figure 1:**
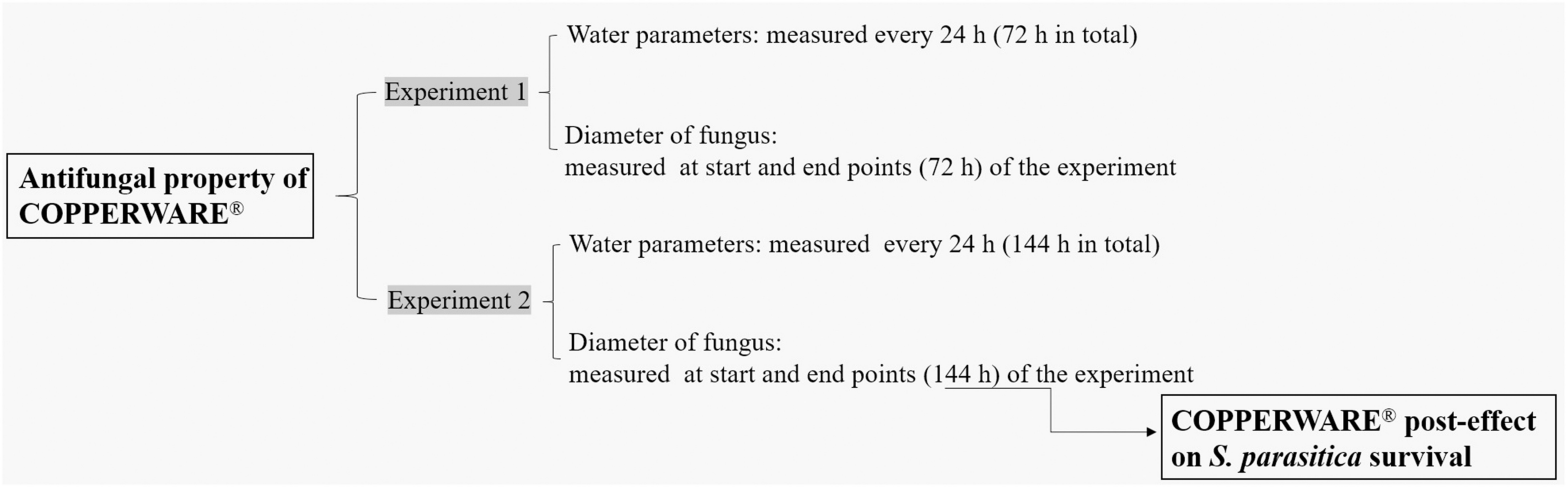
The flow chart of the CCCSNs filter (COPPERWARE^®^) filtration experiments.

To determine whether *S. parasitica* was killed or simply inhibited by COPPERWARE^®^, agar plugs from tanks in experiment 2 were removed at the end of the study and transferred to a new PDA medium without CCCSNs (Figure 1). The initial diameter of the *S. parasitica* was identified as the diameter measured at the end of experiment 2 (144 h). Agar plugs were incubated at 25°C for 72 h and measured every 24 h. The growth of the plugs post exposure to COPPERWARE^®^ was compared over time (refer to section Statistical Analyses).

### Water Quality

Dissolved oxygen and pH were measured in the tanks at 24 h intervals using probes (YSI ProODO, Xylnm, USA) during the filtration experiments (experiment 1 and experiment 2). To determine water turbidity, optical densities (OD_600_) were measured every 24 h with a spectrophotometer (Biophotometer, Eppendorf, Germany). We assessed the levels of copper (ionized and bound), ammonia, nitrite, and nitrate in the water at the end of the experiments using HACH test kits (HACH Inc. Loveland, Colorado). We compared the water quality parameters across treatment groups over time (refer to section Statistical Analyses).

### Statistical Analyses

To compare the potential effects of treatments on the growth of the agar plugs over time (repeated measurements) we used mixed-effects linear regression models controlling for time. Data from each experiment were analyzed separately. The level of significance was set at 0.05. All statistical analyses were performed in GraphPad Prism software (Version 8.0.1, GraphPad Software, San Diego, USA). In brief, we compared the growth of mycelium plugs (diameter in cm) between treatment groups (10 groups for CCCSNs powder experiment, three groups for filtration experiment 1 and 2, and the post-effect evaluation) over time (5 time points for CCCSNs powder experiment; 2 time points for filtration experiments 1 and 2; and 4 time points for the post-effect evaluation). The main effects and the interaction terms between treatments and time points were included in the models as fixed effects, and the individual plates or tanks were included as random effects to account for the repeated measurements within each of the plates or tanks (depending on the experiment) over time.

Water quality parameters were also compared between the treatment groups in a similar manner using mixed-effects linear regression models. Tukey's HSD test was used following the regression models in the case of multiple comparisons.

## Results

### CCCSNs on the Growth of Mycelium

**Figure 2:**
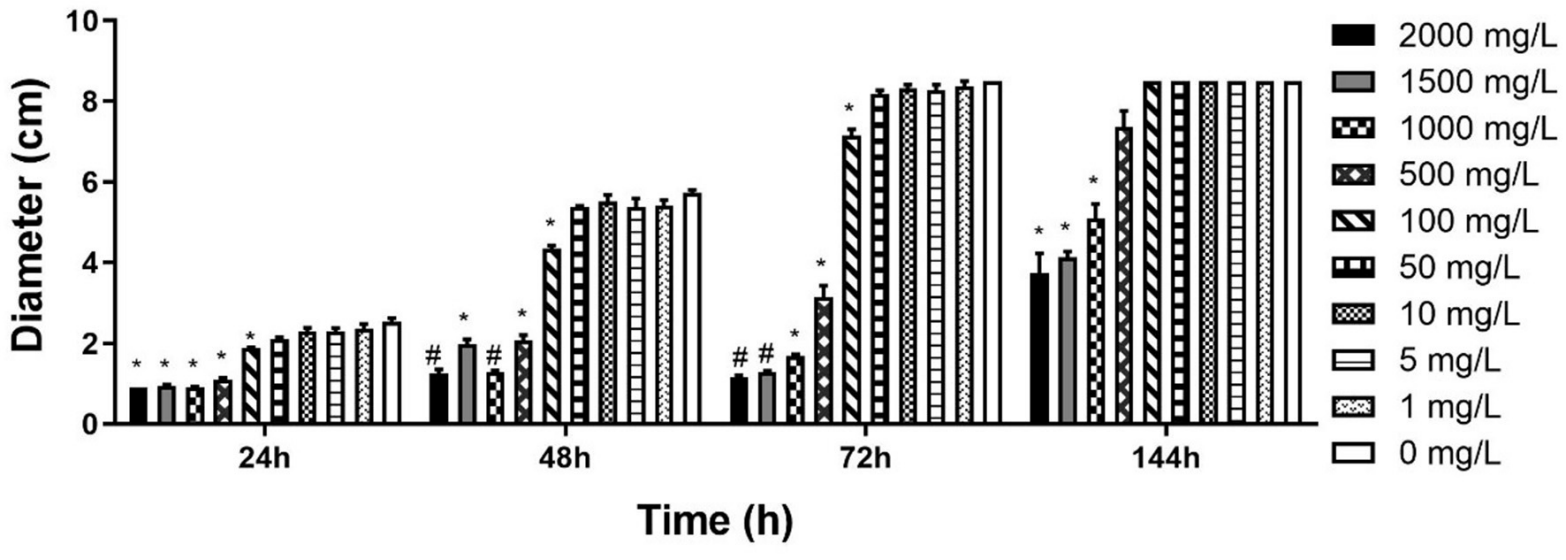
The diameter of *S. parasitica* at different timepoints after treating with different concentrations CCCSNs. *: 0.001 < *p* < 0.05, #: *p* < 0.001. Error bars are ± SD, *n* = 3.

CCCSNs inhibited the growth of *S. parasitica*, and this effect appeared to be dose-dependent (Figure 2 and Supplementary Figure 1). Compared with the control group, *S. parasitica* mycelia was significantly reduced in the presence of CCCSNs at concentrations of 2,000, 1,500, and 1,000 mg/L. All three of these concentrations of CCCSNs significantly inhibited the growth of *S. parasitica* at all experimental time points (*p* < 0.05). The lower concentrations of CCCSNs (i.e., 500 and 100 mg/L) only significantly inhibited the growth for the first 3 days (Figure 2).

### COPPERWARE^®^ Filtration Experiments

**Figure 3:**
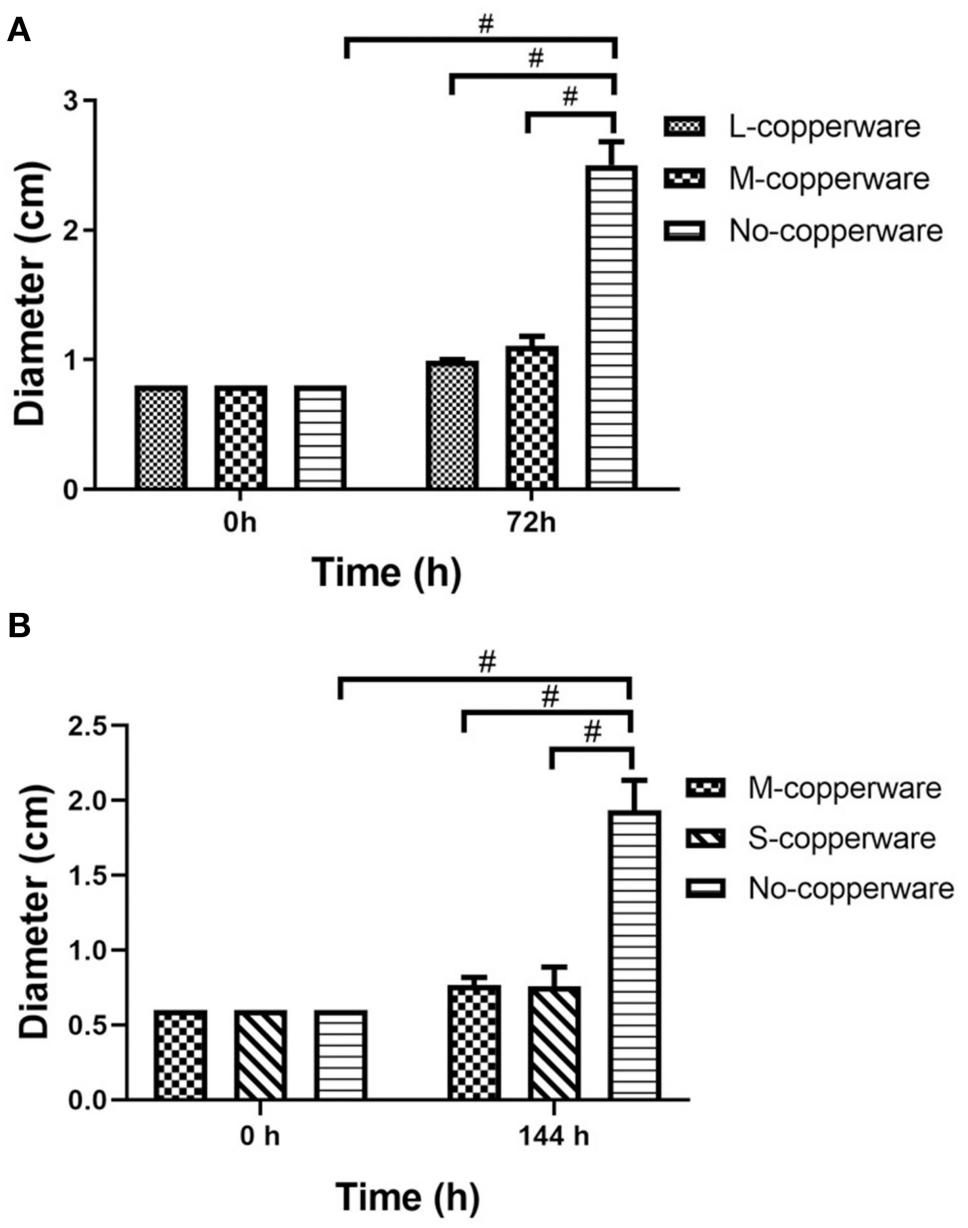
Diameter of *S. parasitica* after water treatment with different quantities of COPPERWARE^®^ filters (L-copperware: 7.5 × 3.7 × 2.4 cm, 9.46 g; M-copperware: 7.5 × 3.7 × 1.2 cm, 4.96 g; S-copperware: 3.75 × 3.7 × 1.2 cm, 2.58 g) and commercial filter with no CCCSNs (No-copperware) in **(A)** experiment 1 and **(B)** experiment 2. #: *p* < 0.001. Error bars are ± SD, *n* = 3.

COPPERWARE^®^ inhibited the growth of *S. parasitica* in aquarium water regardless of the quantity of material used in our filters (Figure 3 and Supplementary Figure 2). At the end of the exposure, the size of agar plugs in the treatment groups was significantly smaller than those of the control group in experiments 1 and 2 (*p* < 0.001) (Figure 3).

**Figure 4:**
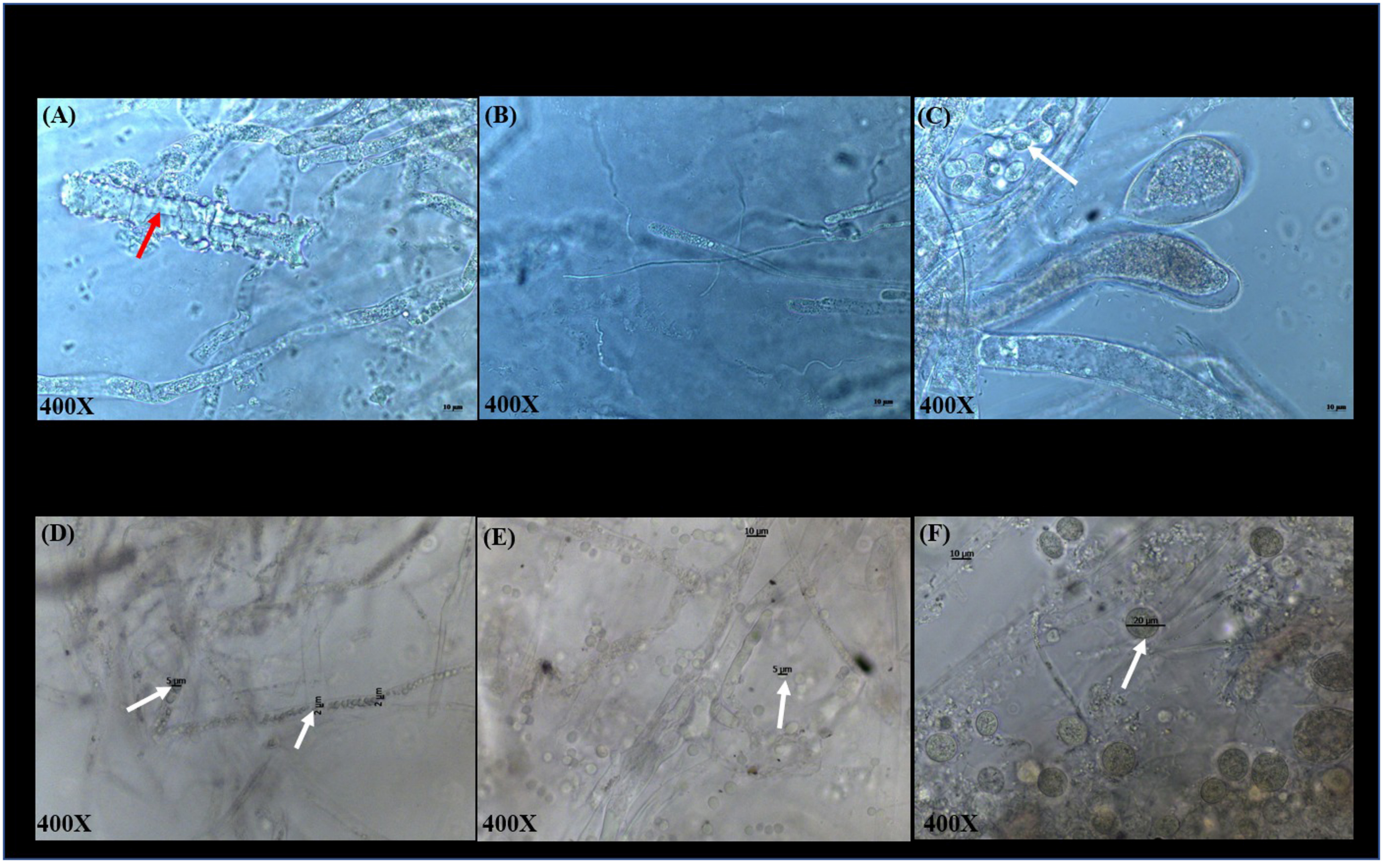
Microscopic observations of *S. parasitica* after treating tanks with different quantities of COPPERWARE^®^ (L-copperware: 7.5 × 3.7 × 2.4 cm, 9.46 g; M-copperware: 7.5 × 3.7 × 1.2 cm, 4.96 g; S-copperware: 3.75 × 3.7 × 1.2 cm, 2.58 g) and commercial filter with no CCCSNs (No-copperware) in experiment 1 at 72 h **(A–C)** and experiment 2 at 144 h **(D–F)**. red arrow: damaged hyphae; White arrow: spores. The spore size in **(D)**: 2–5 μm; The spore size in **(E)**: around 5 μm; The spore size in **(F)**: around 20 μm.

Microscopic examination of *S. parasitica* following exposure to COPPERWARE^®^ revealed morphological changes in the hyphae (Figure 4). In experiment 1, *S. parasitica* treated with COPPERWARE^®^ in both the L-copperware and M-copperware groups displayed some damaged hyphae compared to the non-treated group. The size of spores in the COPPERWARE^®^ treated and untreated groups may have also been affected. We observed that treated *S. parasitica* commonly had spores with diameters ranging between 2 and 5 μm and untreated *S. parasitica* had spores ~10-fold larger in diameter at 20 μm (Figure 4).

### Post-exposure Effect of COPPERWARE^®^ on *S. parasitica* Survival

**Figure 5:**
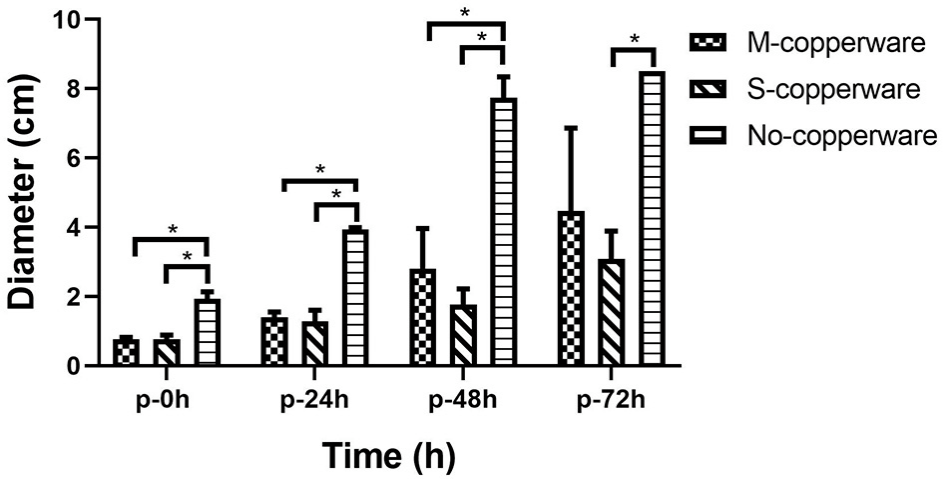
Post-effect of different quantities of COPPERWARE^®^ (M-copperware: 7.5 × 3.7 × 1.2 cm, 4.96 g; S-copperware: 3.75 × 3.7 × 1.2 cm, 2.58 g; No-copperware: control group with no CCCSNs in the filter) on the development of *S. parasitica*. *: 0.001 < *p* < 0.05. *p*: post effect after the removal of the COPPERWARE^®^. Error bars are ± SD, *n* = 3.

Once the treatment (exposure to COPPERWARE^®^) was halted, the mycelium growth remained minimal for the first 24 and 48 h compared to the non-treated control group (*p* < 0.05). However, after 72 h, the mycelium plugs appeared to increase in size. Only one treated group (S-copperware) still had a statistically smaller agar plug relative to the control systems by the end of the study (Figure 5 and Supplementary Figure 3).

### Water Quality

Copper was detected in the COPPERWARE^®^ treated groups, and the range of copper concentration based on the HACH test was higher for tanks treated with larger quantities of the product (Table 2). The concentration of copper in the water was as high as the detection limit of our test kits (3 mg/L) in the treatment group with the largest quantity of COPPERWARE^®^ (L-copperware: 7.5 × 3.7 × 2.4 cm, 9.46 g). Concentrations of dissolved oxygen, ammonia, nitrite, and nitrate were all within the normal range for all treatment groups (Supplementary Figure 4 and Supplementary Table 1). However, at the end of experiment 1, the pH in the L-copperware treated group was significantly lower than the control (*p* < 0.05) (Supplementary Figure 4).

**Table 2:** Copper concentration after water treatment with different sizes of COPPERWARE^®^ filters at the end of experiments 1 and 2.

**Experiment**	**Treatment^a^**	**Copper (mg/L)**
Experiment 1 (72 h)	L-copperware	≥3
	M-copperware	1–3
	No-copperware	0
Experiment 2 (144 h)	M-copperware	1–3
	S-copperware	0.5–1
	No-copperware	0

a *L-copperware: 7.5 × 3.7 × 2.4 cm, 9.46 g; M-copperware: 7.5 × 3.7 × 1.2 cm, 4.96 g; S-copperware: 3.75 × 3.7 × 1.2 cm, 2.58 g; and No-copperware: commercial filter with no CCCSNs*.

**Figure 6:**
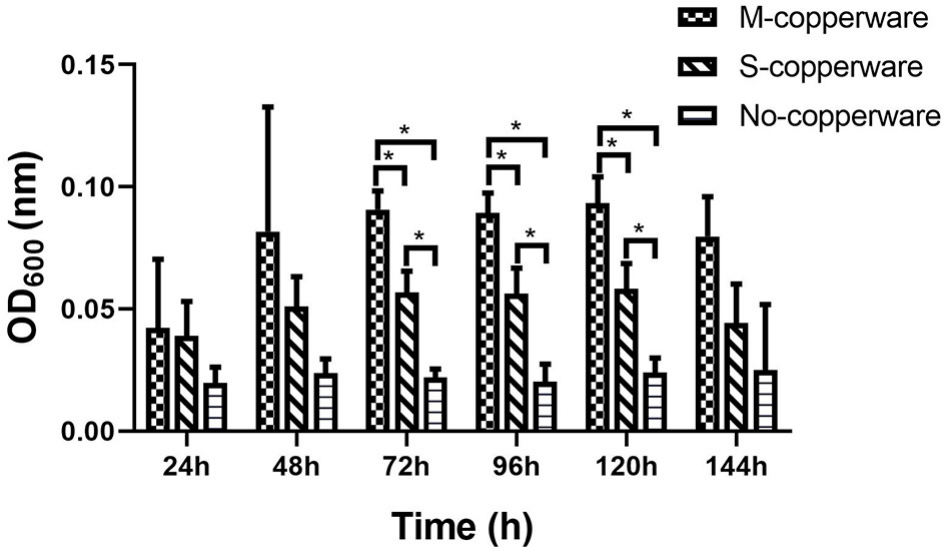
Turbidity of water in experiment 2 assessed via spectrophotometer. M- copperware: 7.5 × 3.7 × 1.2 cm, 4.96 g; S-copperware: 3.75 × 3.7 × 1.2 cm, 2.58 g; No-copperware: control group with no CCCSNs in the filter. *: 0.001 < *p* < 0.05. Error bars are ± SD, *n* = 3.

The turbidity of water changed over time in the treatment tanks. After 72 h, 96 h and 120 h of exposure to COPPERWARE^®^, OD_600_ measurements water in the treated groups were significantly higher compared to the control (*p* < 0.05) (Figure 6). This increase in turbidity was particularly obvious in tanks treated with the medium quantity of product (i.e., M-copperware 7.5 × 3.7 × 1.2 cm, 4.96 g) (Supplementary Figure 5). The OD_600_ measurements of tanks treated with M-copperware were significantly higher than measurements in control tanks, as well as tanks treated with the smallest quantity of COPPERWARE^®^ (labeled S-copperware with 3.75 × 3.7 × 1.2 cm, 2.58 g) (*p* < 0.05) (Figure 6) between 72 and 120 h. Although the water turbidity was still higher in the treated tanks than the control tanks by 144 h post-exposure, this difference was no longer statistically significant (Figure 6).

## Discussion

Based on the results of this study, it appears that CCCSNs and its commercial filter product (COPPERWARE^®^) may inhibit the growth of *S. parasitica*. The dose-dependent suppression of the growth of this organism (i.e., minimal growth in the agar plug size) in our experiments (Figures 2, 3) was also supported by morphological evidence of damage to the hyphae and spores (Figure 4); however, the effect of CCCSNs on the growth did not persist more than a few days once we removed the filter material from the aquarium (Figure 5). This suggests continuous exposure or intermittent exposure to the CCCSNs may be required to maintain *S. parasitica* at a minimal level in aquatic environments.

The dose-dependent disinfection activity of copper nanoparticles has also been reported by others with fungal species (47, 48). Removal of pathogenic *Fusarium* species affecting plants has been demonstrated with copper nanoparticles (36). The antifungal properties of copper nanoparticles were reported to be better than other metal-based nanoparticles (Ag, Zn, and Au nanoparticles), as well as the commercial fungicide containing Cu(OH)_2_ (49).

Copper nanoparticles have a large surface-to-volume ratio which may enhanced antimicrobial efficiency (40). The mechanism of action of nanoparticles on microbes is not well-established, but the disinfection properties may be through a direct metal-microbial contact mechanism, which is enhanced by the large surface area to the volume of the nanoparticles (50–52). It is also possible in our study that free copper ions were released and played a role in the inhibition of *S. parasitica* growth, as we measured copper in the water after the treatment. This has been reported to be one of the microbial killing mechanisms of metal-based nanoparticles (53, 54). Direct contact with copper ions causes a decline in the membrane integrity of microbes, leading to a subsequent leakage of cell contents and eventually cell death (51, 55). Copper ions can also penetrate the cell, leading to lipid peroxidation, protein oxidation and DNA damage (17, 56, 57).

We observed a dose-dependent increase in turbidity during experiment 2 (Figure 6), which could potentially have been related to a stress response sporulation event. Although we could not verify this phenomenon, Kasprowicz et al. (58) reported an increase in the release of spores from *Fusarium culmorum* associated with exposure to silver nanoparticles. Spores are better adapted to survive harsh environmental conditions with their thick cell walls compared to the vegetative cell walls (59). Spores are resistant to many types of environmental stresses such as starvation (60) and heavy-metal exposure (61). It is important to note that the quantity of *S. parasitica* used in this laboratory experiment was high relative to the volume of water. It is possible that under natural levels of the pathogen, water turbidity would not be noticeably affected even in the event of sporulation.

Interestingly, if sporulation was the reason for the turbidity it did not lead to better germination or mycelial growth, suggesting that successful inhibition on *S. parasitica* in aquatic systems can be achieved in the presence of CCCSNs. The precise concentration of CCCSNs required for inhibition of *S. parasitica* growth in larger aquaculture systems remains to be determined, but this study could be used as a starting point to assess the economic value of incorporating this material into filtration systems. The duration of activity of CCCSNs in a system also needs to be established, and this may be of critical importance because once we removed the COPPERWARE^®^ we eventually observed renewed growth of the oomycete. The fact that *S. parasitica* was able to grow once we removed the agar plugs from the COPPERWARE^®^-challenged water was unfortunate, but not unexpected given the resilience of *Saprolegnia* spp. spores to environmental insults (62). Microscopic evaluation of the oomycete pathogens after exposure to CCCSNs showed that the spores remained intact and within the hyphae even though they were smaller in size than the controls (Figure 4). The persistence of spores in hyphae may have enabled the rejuvenation of *S. parasitica* when transferred from COPPERWARE^®^-challenged tanks to a copper-free environment (Figure 5 and Supplementary Figure 3). Zinc oxide nanoparticles have also been shown to be fungistatic rather than fungicidal at certain concentrations against *F. graminearum* (63).

The continuous use of COPPERWARE^®^ in the filtration systems at the highest concentration (L-copperware) in our study resulted in high levels of total copper in the water (i.e., above the test detection limit of 3 mg/L). Although the test kit we used measured both copper ions and bound copper, it was concerning to have this level of copper leaching from the filter material. Fortunately, this quantity of COPPERWARE^®^ was not necessary for disinfecting water; we still observed disinfection when we used lower levels of material in our aquaria. However, even using the smallest quantity of COPPERWARE^®^ (S-copperware) we were able to detect low levels of total copper (0.5–1 mg/L). This level of material was within the safe range of drinking water (64), but could still be toxic to some aquatic animals. For example, the 48-h LC50 values of copper sulfate for *Macrobrachium lamarrei* and *Macrobrachium dayanum* were 0.361 and 0.988 mg/L, respectively (65), while the 48-h LC50 for fed neonates of *Daphnia magna* was 18.5 μg/L (66). However, for species like tilapia (*Oreochromis niloticus*), and catfish (*Clarias gariepinus*), the 96-h LC50 values are approximately 58.8 and 70.1 mg/L, respectively (67), which is higher than what we detected in this study using the lowest concentration of COPPERWARE^®^.

It is not possible to evaluate the toxicity of COPPERWARE^®^ by the measured estimated copper concentration in this study as it may not have all been free copper ions. If materials constructed from CCCSNs are to be explored for use in aquaculture, further research is needed to reduce or prevent copper released from nanoparticles to avoid copper toxicity. This advice has also been advocated by other researchers (47). The other water quality parameters monitored in this study were not negatively impacted by the use of COPPERWARE^®^.

The potential leaching of copper from the commercial CCCSN material tested in this study may be reduced by improving the coating of the nanoparticles on the filtration material. Given this issues is rectified, our study indicates that CCCSNs could be quite effective at controlling *S. parasitica* and is worth further investigation. Further research and animal safety assessments are required before this technology can be scaled up to large aquaculture systems, but the initial findings of this study are promising if the CCCSN can remain attached to the filter material more effectively.

## Data Availability Statement

The original contributions presented in the study are included in the article/Supplementary Material, further inquiries can be directed to the corresponding author/s.

## Author Contributions

JZ and SS-H came up with the idea of the study. JC and QH assisted with data acquisition. HL, PJ, and JW provided the stored *S. parasitica* and essential equipment for experimenting, as well as guidance in the laboratory for the culture of the pathogen. NL assisted with the analysis of *S. parasitica* morphology. BM, LH, YY, and PN were involved in manuscript revising. ON contributed to the statistical analysis. JZ conducted most of the studies and wrote the manuscript with the assistance and comments from JC, QH, BM, ON, HL, PJ, JW, NL, LH, YY, PN, and SS-H. All authors contributed to the article and approved the submitted version.

## Conflict of Interest

The authors declare that the research was conducted in the absence of any commercial or financial relationships that could be construed as a potential conflict of interest.

## Publisher's Note

All claims expressed in this article are solely those of the authors and do not necessarily represent those of their affiliated organizations, or those of the publisher, the editors and the reviewers. Any product that may be evaluated in this article, or claim that may be made by its manufacturer, is not guaranteed or endorsed by the publisher.
