# Supplementary material for: Copper/Carbon Core/Shell Nanoparticles: A Potential Material to Control the Fish Pathogen Saprolegnia parasitica
Source: Front Vet Sci. 2021 Jul 23;8:689085. doi: 10.3389/fvets.2021.689085 (PMC8342997; doi:10.3389/fvets.2021.689085)
Supplement: Supplementary file 1 [file Data_Sheet_1.PDF]

Supplementary information

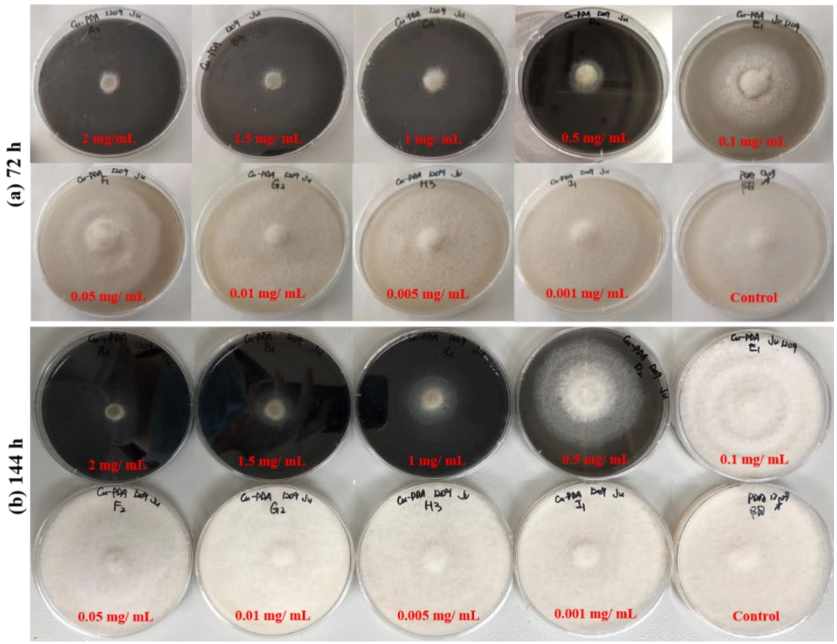

Supplementary Figure 1. Growth of *Saprolegnia parasitica* on PDA plates with the presence of different concentrations of CCCSNs at (a) 72 h and (b) 144 h.

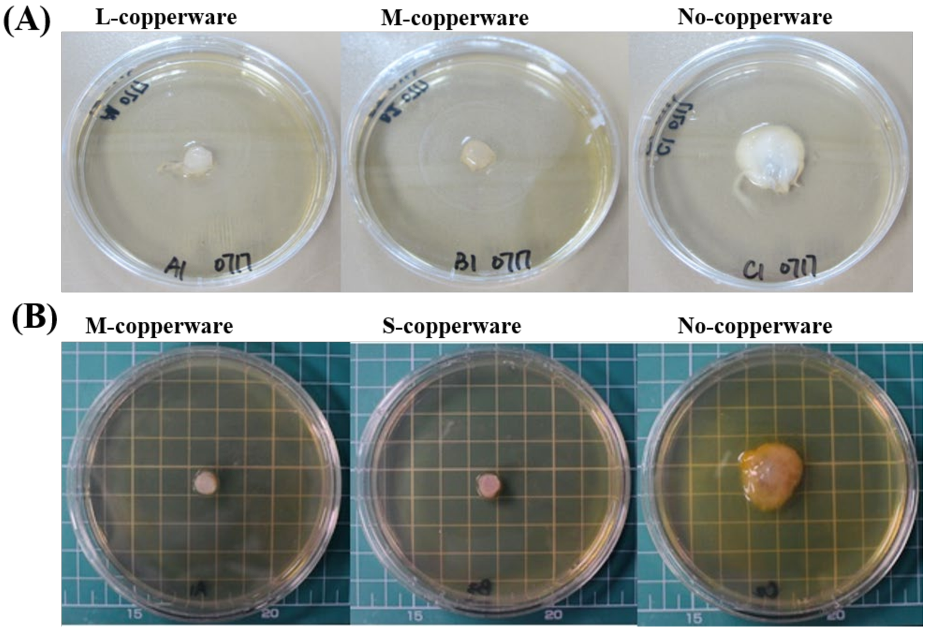

Supplementary Figure 2. Appearance of *S. parasitica* after treating with different quantities of COPPERWARE® (L-copperware: 7.5×3.7×2.4 cm, 9.46 g; M-copperware: 7.5×3.7×1.2 cm, 4.96 g; S-copperware: 3.75×3.7×1.2 cm, 2.58 g) and commercial filter with no CCCSNs (No-copperware) in (A) experiment 1 at 72 h and (B) experiment 2

at 144 h.

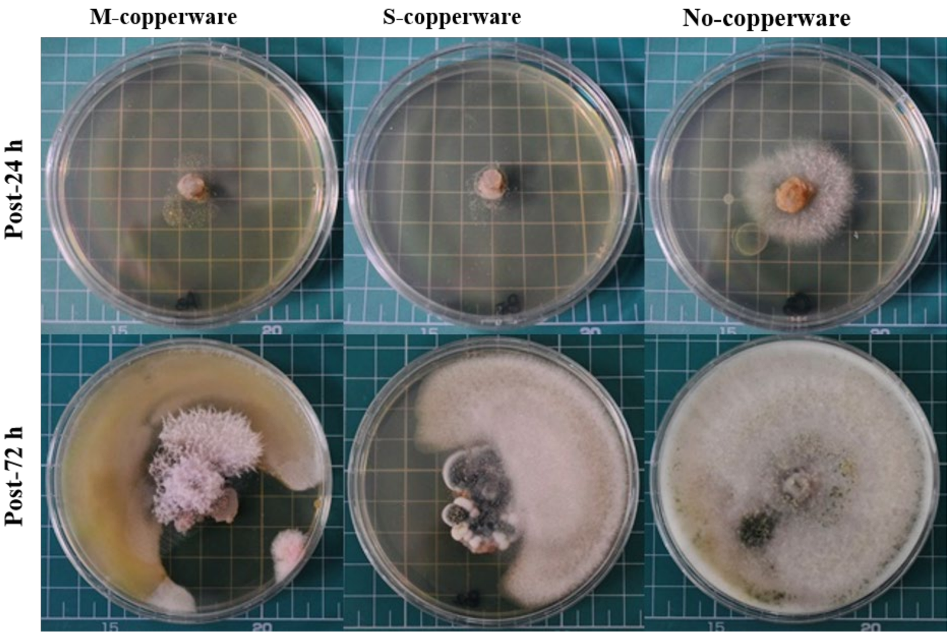

Supplementary Figure 3. Appearance of the post-effect of COPPERWARE® on the development of *S. parasitica*. M-copperware: 7.5×3.7×1.2 cm, 4.96 g; S-copperware: 3.75×3.7×1.2 cm, 2.58 g; and No-copperware: control group with no CCCSNs in the filter.

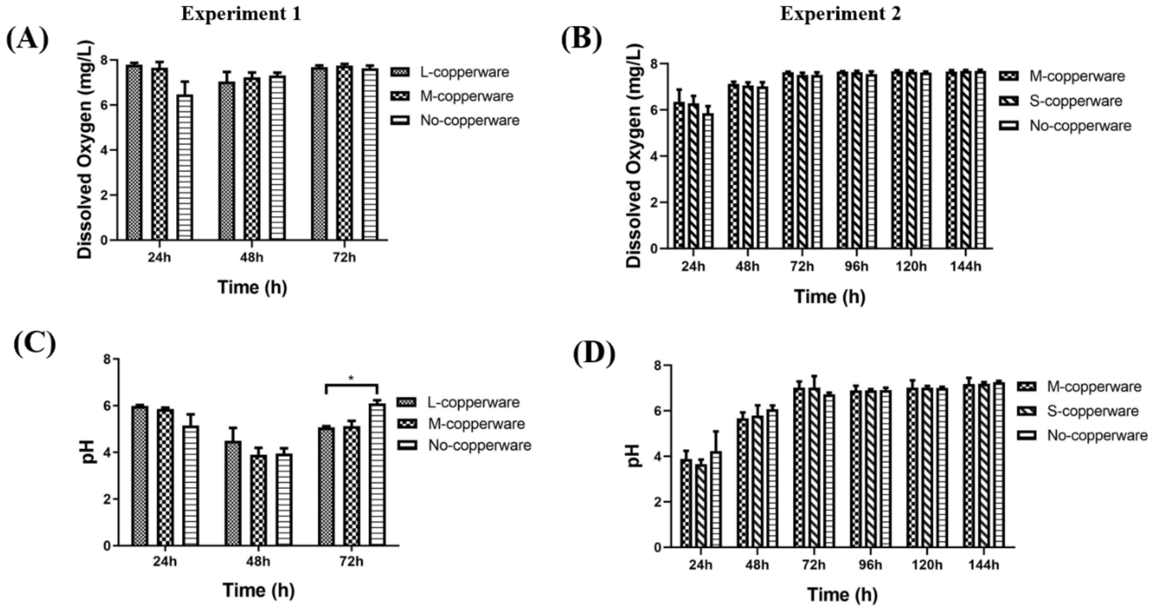

Supplementary Figure 4. Water quality (dissolved oxygen: A&B and pH: C&D) in experiments 1 and 2. L-copperware: 7.5×3.7×2.4 cm, 9.46 g; M-copperware: 7.5×3.7×1.2 cm, 4.96 g; S-copperware: 3.75×3.7×1.2 cm, 2.58 g; No-copperware: control group with no CCCSNs in the filter. *: $0.001 < p < 0.05$. Error bars are ± SD, n=3.

Supplementary Table 1. Water quality (ammonia, nitrite, and nitrate) at the end of time point

Experiment	Treatment	Ammonia (mg/L)	Nitrite (mg/L)	Nitrate (mg/L)
Experiment 1 (72)	L-copperware	Below detection limit		
	M-copperware	Below detection limit		
	No-copperware	Below detection limit		
Experiment 2 (144 h)	M-copperware	Below detection limit		
	S-copperware	Below detection limit		
	No-copperware	Below detection limit		

Note: L-copperware: 7.5×3.7×2.4 cm, 9.46 g; M-copperware: 7.5×3.7×1.2 cm, 4.96 g; S-copperware: 3.75×3.7×1.2 cm, 2.58 g and No-copperware: commercial filter with no CCCSNs.

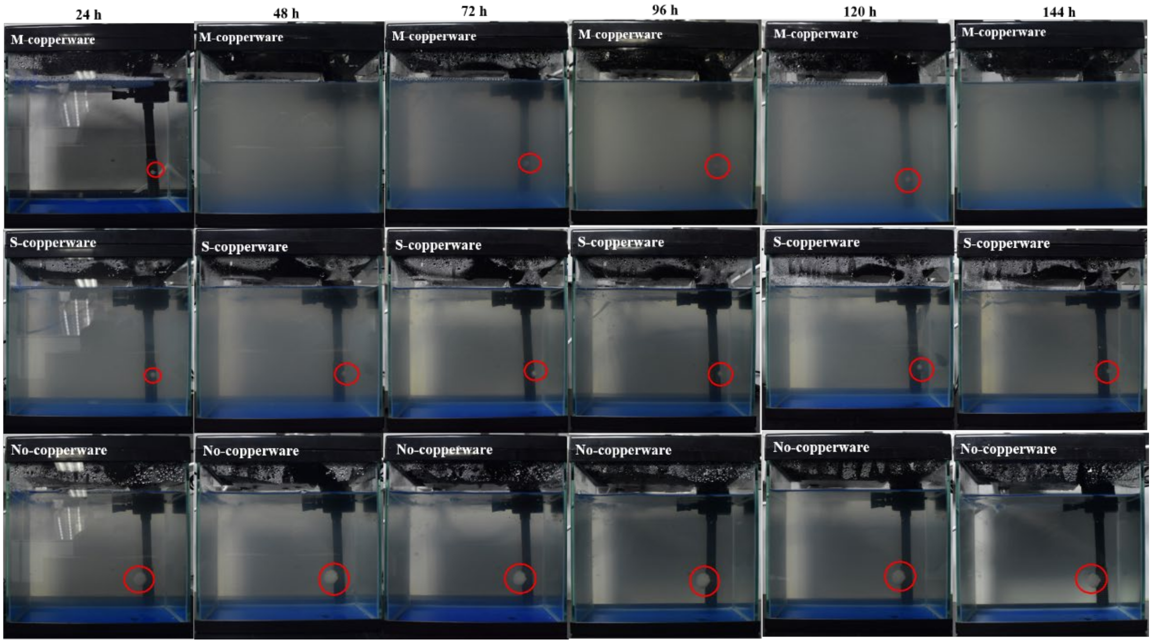

Supplementary Figure 5. Water transparency and appearance of *S. parasitica* after treating with different quantities of COPPERWARE® in experiment 2. M-copperware : 7.5×3.7×1.2 cm, 4.96 g; S-copperware: 3.75×3.7×1.2 cm, 2.58 g; and No-copperware: control group with no CCCSNs in the filter.
